# Supersaturated state of diazepam injection following dilution with infusion fluid

**DOI:** 10.1186/s40780-014-0009-9

**Published:** 2015-03-09

**Authors:** Yoshinori Onuki, Naoki Hasegawa, Chihiro Kida, Mayumi Ikegami-Kawai, Masayoshi Tsubuki, Shunsuke Shirozu, Yasuko Obata, Kozo Takayama

**Affiliations:** Department of Pharmaceutics, Hoshi University, 2-4-41 Ebara, Shinagawa, Tokyo 142-8501 Japan; Central Research Laboratories, Hoshi University, 2-4-41 Ebara, Shinagawa, Tokyo 142-8501 Japan; Division of Applied Pharmaceutical Education and Research, Hoshi University, 2-4-41 Ebara, Shinagawa, Tokyo 142-8501 Japan

**Keywords:** Diazepam injection, Supersaturated state, Infusion fluids, Precipitation, IR microscopy, 1H-NMR

## Abstract

**Background:**

Significant precipitation produced by the dilution of diazepam (DZP) injection with an infusion fluid is a great concern for the clinical practice. In this study, the precipitation behavior under different conditions was investigated.

**Method:**

For the sample preparation, DZP injections (Horizon injection and Cercine injection) were diluted with various infusion fluids (Saline, 5% glucose infusion fluid and Soldem 3A) at designated dilution ratios ranging from 1× to 40× (5 mg/mL to 0.125 mg/mL). In addition, to measure the solubility of DZP in the samples, the saturated solutions of DZP were prepared. The DZP concentrations in the samples were measured by high-performance liquid chromatography (HPLC). This study also investigated the precipitate using various analytical methods: infrared microscopy, ^1^H-NMR, differential scanning calorimetry, and powder X-ray deflection.

**Results:**

First, the compatibility of injection with infusion fluids was investigated. Significant precipitation occurred at dilution ratios ranging from 2× to 20×. No significant effects of formulations and infusion fluids on the compatibility were observed. The solubility of DZP was then further investigated. The concentration of DZP dissolved in the admixtures was higher than the solubility. This indicated that DZP existed in a supersaturated state in the infusion fluid admixtures. In the next phase of this study, the precipitate was investigated using various analytical methods. Results showed that the precipitate in infusion fluid admixtures was mostly composed of DZP, but also contained small amounts of the ingredients of DZP injection, such as benzoic acid and benzyl alcohol.

**Conclusions:**

This study clarified details of the precipitation occurring after dilution of DZP injection with infusion fluids. It is worth noting that DZP in an infusion admixture existed in a supersaturated state. These findings offer important insight into the clinical practice of DZP injection.

## Background

Diazepam (DZP), a type of benzodiazepine drug, has sedative and muscle relaxant effects. The indications of DZP injection are as follows: tension and anxiety, repeated or prolonged epileptic seizures, muscle spasm, muscle stiffness due to cerebral palsy or paraplegia, to help relax patients before an operation (preoperative medication), to relieve symptoms of alcohol withdrawal, and others. Due to its ease of use, safety, rarity of allergic reactions, and the production of varied degrees of desirable amnesia, DZP injection has been widely used in clinical practice.

DZP is a very poorly water soluble drug; thus, the injectable dosage form contains a considerable amount of organic solvent, namely 40% (v/v) propylene glycol and 10% (v/v) ethanol. With regard to the clinical practice of DZP injection, there are several things to consider. First, it is well known that the dilution of DZP injection with an infusion fluid results in significant precipitation; the dilution immediately produces a light yellow to white insoluble precipitate. Therefore, in the drug package insert, there is a warning that the injection should not be mixed or diluted with other solutions or drugs and not added to intravenous fluids.

Despite the manufacturer’s instructions, in practice there is a great demand for the administration of diluted DZP injection via continuous i.v. injection. It is known that the precipitate produced is redissolved by further dilution; at a high dilution ratio, the infusion fluid admixture appears to be clear. This feature enhances the possibility that diluted DZP injection can be administered without concern for the precipitation.

A number of studies have been carried out on the compatibility of DZP injection with infusion fluids. Jusko et al. reported no evidence of DZP precipitation above a dilution ratio of 15× (0.3 to 0.4 mg/mL) [1]. According to Morris, DZP injection at a dilution of 40× (0.12 mg/mL) or greater is visually compatible and chemically stable in 5% dextrose, Ringer’s injection, lactated Ringer’s injection, and saline for 24 h [2]. Tehrani and Cavanaugh found no substantial cloudiness after dilution of DZP injection with lactated Ringer’s injection at a dilution ratio of 25× (0.2 mg/mL) [3]. Furthermore, there were no visible precipitates in the DZP injections diluted with saline [4] and 5% dextrose [5] at a dilution ratio of 50× (0.1 mg/mL). Despite the best endeavors to date, a clear answer has yet to be found to the question of how to dilute the DZP injection for safe clinical practice. One reason for this is that all evidence to date lacks an objective viewpoint. This is because these studies were mostly performed using visible inspection. We speculated that the subjective bias of visible inspection may prevent consensus being reached.

If we can determine the dilution conditions under which precipitation does not occur, we can establish a suitable procedure for diluting DZP injection with infusion fluids for clinical practice. The purpose of this study was to investigate precipitation occurring after the dilution of DZP injection with infusion fluids. We first investigated the compatibility of DZP injection with infusion fluids. In the course of our experiments, we found that DZP in the diluted injections existed in a supersaturated state. We then analyzed the precipitate collected from the diluted injections in detail. The results of this study should provide valuable information on the clinical practice of DZP injection.

## Methods

### Materials

DZP injections (Horizon injection and Cercine injection) were purchased from Maruishi Pharmaceutical Co. (Osaka, Japan) and Takeda Pharmaceutical Co. (Osaka, Japan), respectively. Saline (Otsuka normal saline) and 5% glucose infusion fluid (Otsuka glucose injection 5%) were purchased from Otsuka Pharmaceutical Co. (Tokyo, Japan). Soldem 3A (glucose-electrolyte solution) was purchased from Terumo (Tokyo, Japan). DZP was purchased from Wako (Osaka, Japan). All other chemicals were of analytical grade and commercially available.

### Measurement of DZP in the infusion fluid admixtures

For sample preparation, marketed DZP injections (Horizon injection and Cercine injection) were diluted with various infusion fluids at designated dilution ratios ranging from 1× to 40× (5 mg/mL to 0.125 mg/mL). The diluted DZP injection was passed through a 0.2 μm inline filter (Terufusion final filter PS; Terumo, Tokyo, Japan), and then the filtrate was mixed with the designated volume of the mobile phase (acetonitrile/water, 50:50 v/v) to dissolve DZP completely in it. Afterwards, the DZP concentration in the filtrate was measured by high-performance liquid chromatography (HPLC). The sample solution was injected into a Hitachi L-2130 HPLC pump equipped with a C18 reversed-phase column (YMC-pack A-302 S-5 A, 150 × 4.6 mm i.d.; Yamamura Chemical Laboratories, Kyoto, Japan). A Hitachi UV detector L-2400 (Tokyo, Japan) was set at 254 nm. Acetonitrile/water (50:50 v/v) was used as the mobile phase, the flow rate was 1.0 mL/min, and HPLC analysis was performed at room temperature. EZChrom Elite Chromatography Data System (Hitachi, Tokyo, Japan) was used as the acquisition and analysis software.

### Nuclear magnetic resonance (NMR) study

After dilution of DZP injection with water, the precipitate was separated from the supernatant by centrifugation at 13,000 *g* for 5 min, dried in the open air, and then dissolved in CD_3_OD. As a control, analytical-grade DZP was dissolved in CD_3_OD. All NMR measurements were performed using a JEOL JMN-LA500 spectrometer (^1^H at 500 MHz, 11.7 T) at 30°C. ^1^H chemical shifts were referenced to those of the external standard tetramethylsilane (*δ* = 0 ppm).

### Powder X-ray diffraction (PXRD) measurements

Precipitate was collected from diluted DZP injection by centrifugation, as described above. After drying the collected precipitate, the PXRD pattern was acquired using a SmartLab X-Ray diffractometer (Rigaku, Tokyo, Japan). Furthermore, the PXRD pattern of analytical-grade DZP was acquired for comparison with the crystal form.

### Differential scanning calorimetry (DSC) measurement

The dried precipitate and analytical-grade DZP were placed in an aluminum pans (Rigaku, Tokyo, Japan). The DSC measurements were performed with a Thermoplus DSC 8230 (Rigaku, Tokyo, Japan), with heating scans at a rate of 1°C/min.

### Infrared (IR) microscopy measurement

Fourier transform infrared spectroscopy (FTIR) spectra of precipitate and analytical-grade DZP were recorded on a JASCO FT/IR-4200 type A spectrometer using the reflection method. The FTIR spectra were measured over the range 4000–650 cm^−1^ with resolution of 4 cm^−1^ for 16 scans.

## Results and discussion

**Figure 1:**
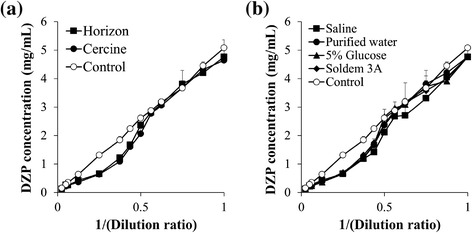
**Precipitation behaviors as a function of (a) marketed products of DZP and (b) infusion fluids. (a)** Horizon injection or Cercine injection was diluted with purified water. **(b)** Horizon injection was diluted with saline, purified water, 5% glucose injection, and Soldem 3A. Horizon injection was completely dissolved in acetonitrile/water (50:50 v/v), and it was used as a control. Each value represents the mean ± S.D., N = 3.

We first investigated the compatibility of the DZP injection with infusion fluids. For sample preparation, diluted DZP injection was passed through a 0.2 μm inline filter to remove precipitate, and then the DZP concentration was determined by HPLC (Figure 1). As a control, we also diluted the Horizon injection with acetonitrile/water (50:50 v/v), in which the DZP was completely dissolved. As shown in Figure 1, the profile of the sample was slightly different to that of the control. In most samples, the DZP concentrations of the sample were obviously lower than those of the control at the dilution ratios ranging from 2× to 20×, indicating that significant precipitation of DZP occurred under the experimental conditions. The precipitation behavior was similar to that from visible inspection [2].

This study also investigated the effects of formulations and infusion fluids on the compatibility. The profiles of Horizon injection and Cercine injection were identical (Figure 1a). According to their packaging inserts, their formulations are almost the same; thus, this result seems to be reasonable. The profiles, as a function of infusion fluids, were similar to each other (Figure 1b), suggesting that the infusion fluid also appears to have little influence on the compatibility of DZP. This result is supported by previous studies [2,6]. For example, Morris compared the compatibility of DZP injection after dilution with various infusion fluids (5% dextrose, saline, Ringer’s injection and lactated Ringer’s injection), and then reported that there was no perceptible difference caused by the difference in the infusion fluids [2]. Mason et al. determined the solubility of DZP in 5% dextrose, lactated Ringer’s injection and saline, and found that there was little change in the solubility of DZP even though the diluents were changed [6]. DZP is a weakly dissociated base with a pKa of 3.3, attributed to deprotonation of its conjugate acid at the 4-position nitrogen atom [7]. When the pH of the solution is below the pKa, the solubility of DZP is changed substantially [6]. However, the pH of commonly used infusion fluids is much higher than the pKa [6], therefore it is to be expected that changing the infusion fluids will have no significant effect on the compatibility of DZP injection.

In the above experiments, we confirmed the dilution conditions under which significant precipitation occurred. We next investigated the solubility of DZP in the diluted injections. The formation of precipitate is thought to be caused by the DZP concentration of the sample exceeding the solubility. Thus, we expected that samples with precipitate have reached saturation, while samples without precipitate have a capacity to dissolve more DZP.

**Figure 2:**
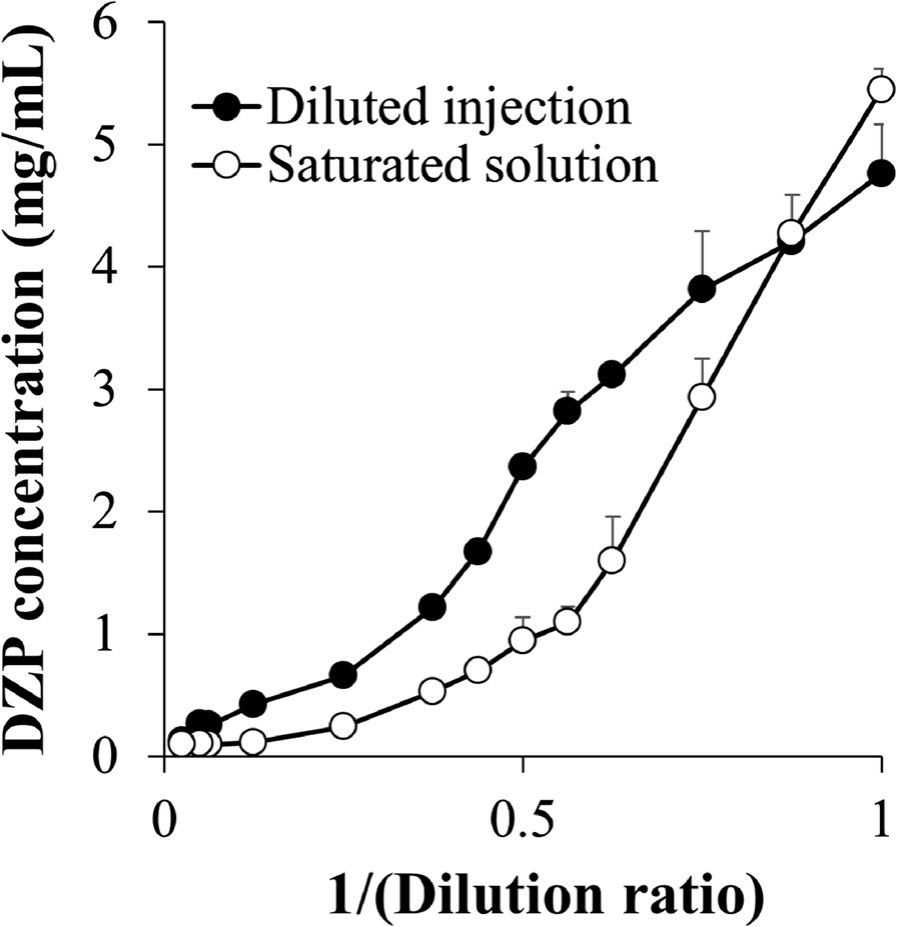
**Solubility curve of DZP in the injection diluted with purified water.** DZP injection was diluted with purified water, in various dilution ratios, at 25°C, after which excessive amounts of DZP powder were added to the DZP injections diluted with water to prepare the saturated solutions. Each value represents the mean ± S.D., N = 3.

For preparation of the saturated solutions, excessive amounts of DZP powder were mixed with DZP injections diluted with purified water. Afterwards, the precipitate and DZP powder were removed by filtration and then their DZP concentrations were measured by HPLC. The observed solubility curve is shown in Figure 2. We note that the solubility of DZP was quite different from our expectation. Except for the original DZP injection (1×), all saturated solutions showed lower DZP concentrations than those of the diluted DZP injections. We also observed similar results from ^1^H-NMR spectra from the samples; compared with the sample without DZP powder, the sample with the powder (saturated solution) showed lower concentration of DZP (data not shown). This result indicates that the infusion fluid admixtures were supersaturated solutions of DZP. We further observed that the DZP concentration was substantially decreased by the addition of metal chip to the diluted DZP injection (data not shown). This is a common feature of supersaturation. Newton et al. also suggested the possibility of supersaturation of DZP [8]. They investigated the solubility of DZP in infusion fluid admixtures. The solubility of DZP in injection at a 1:17.7 dilution ranged from 0.04 to 0.05 mg/mL. On the other hand, when they conducted this study, it had already been reported that the lowest volume ratio that did not result in a precipitate was a 1:40 dilution [2]. They recognized that the DZP concentration at 1:40 dilution was 0.12 mg/mL, and the concentration was higher than the solubility they clarified. They subsequently suggested the supersaturation of DZP in the diluted injection.

For further information, as a preliminary experiment, we evaluated the stability of the supersaturated state of DZP in infusion fluid admixtures. After dilution of DZP injection, we left the sample as it was at room temperature for a few days, and then measured the concentration once again. No change in the DZP concentration was observed (data not shown).

With regard to the stability of DZP in infusion fluid admixtures, there are a number of articles in which this is addressed. For one thing, it is known that DZP is substantially absorbed by polyvinyl chloride (PVC); a substantial reduction in DZP concentration was found after storage of the infusion fluid admixture in a container made of PVC [6,9,10]. In contrast, DZP in the infusion fluid admixtures seems to be stable as long as it is stored in PVC-free containers. For example, there is a report that the DZP concentration in the infusion fluid admixture remained unchanged over a 168 h period when stored in a glass or polyethylene bottle [9]. In our study, all experiments were performed using PVC-free containers (we used polypropylene tubes or glass bottles); thus, it was assumed that the supersaturated state of DZP would be maintained over a long period if the sample was left as is.

In the next phase of the study, we analyzed the precipitate. Jusko et al. have reported that the precipitate comprises almost solely DZP [1]. However, the details of the precipitate had yet to be investigated. We then performed a wide variety of analyses.

**Figure 3:**
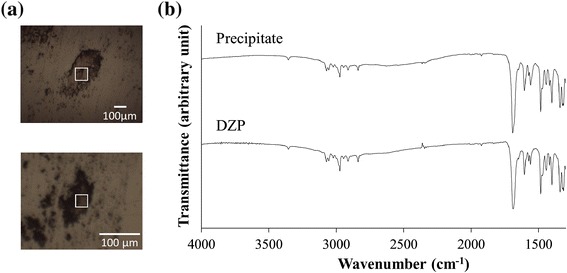
**Microscopic images (a) and FTIR spectra (b) of the precipitate and analytical-grade DZP.** The region of interest for the measurement of the IR spectra is indicated by a square. The aperture sizes used for the precipitate and analytical-grade DZP were 100 μm × 100 μm and 30 μm × 30 μm, respectively. The y-axis of each spectrum has arbitrary units.

**Figure 4:**
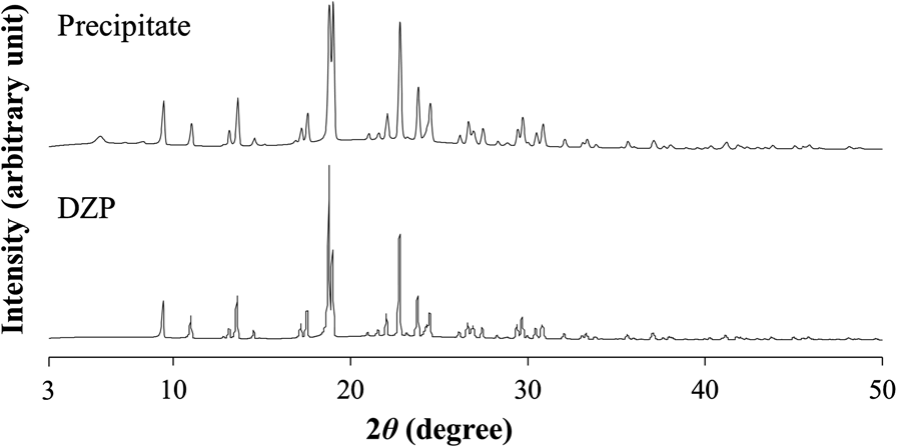
**Powder X-ray diffraction patterns of the precipitate and analytical-grade DZP.**

**Figure 5:**
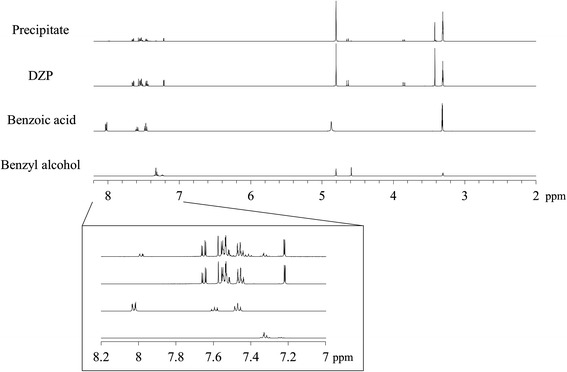
^**1**^ **H-NMR spectra of the precipitate, analytical-grade DZP, benzoic acid and benzyl alcohol dissolved in CD**_**3**_ **OD.**

**Figure 6:**
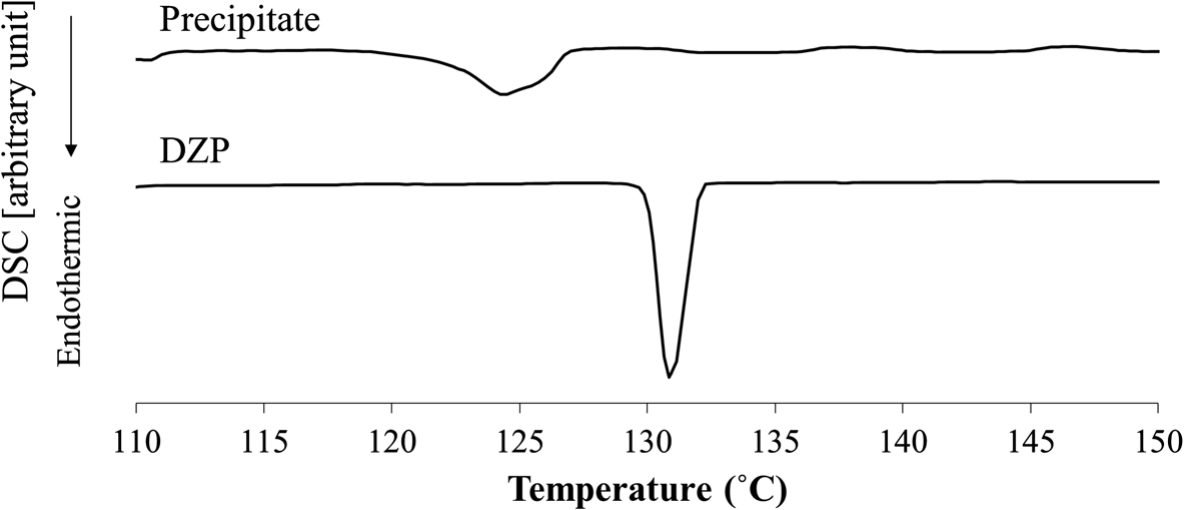
**DSC thermograms of the precipitate and analytical-grade DZP.** The y-axis of each spectrum has arbitrary units.

Figure 3 shows microscopic images and FTIR spectra of the precipitate and analytical-grade DZP. The IR spectra are very similar, indicating that the precipitate was mostly composed of DZP. A little difference was shown in carbonyl stretching region (1800-1700 cm^−1^). The difference may represent the interaction between DZP and some other substances. That is because there is a good possibility that ingredients of DZP injection were coexisted in the precipitate. PXRD patterns of the precipitate appear to be almost the same as for analytical-grade DZP (Figure 4). Thus, in terms of the crystal form, there was no significant difference between the DZP of the precipitate and the analytical-grade DZP. In addition, small peak around 2 theta = 4° were observed. Although the details are still unclear, it probably be derived from ingredients of the DZP injection. We dissolved the precipitate in CD_3_OD, and then acquired the ^1^H-NMR spectrum (Figure 5). Besides the signals for DZP, signals for benzoic acid and benzoic alcohol were also observed, which clarified that some ingredients of DZP injection were present in the precipitate. We also recorded the DSC curve of the precipitate (Figure 6). The endothermic peak corresponding to DZP was obviously lower than in the case of the analytical grade. The depression of the melting point is caused by the coexistence of ingredients in the DZP crystal.

DZP has a wide range of indications; thus, there is a good possibility that the injection is administered to patients simultaneously with other injectable drugs. Much research has been carried out on the compatibility of various injectable products with DZP injection. As far as we know, the injectable drug products are the following: Hextend [11], Precedex [12], Depacon [13], acetaminophen [14], doripenem [15], linezolid [16], and tirofiban hydrochloride [17]. In all the studies, significant precipitation was reported; a white turbid precipitate was observed immediately after the mixing of two injections. All experiments were performed by mixing two injectable products at a 1:1 dilution. Furthermore, all injectable products mixed with DZP injection were water-based injections and free from organic solvent.

Our study revealed that a 1:1 dilution resulted in significant precipitation. The precipitation observed is probably caused by incompatibility of DZP injection with other injectable products. The precipitate comprises mostly DZP.

## Conclusion

This study investigated details of the precipitation occurring after dilution of DZP injection with infusion fluids. DZP in an infusion admixture existed in a supersaturated state. Analysis thereof, using various methods, revealed that the precipitate in infusion fluid admixtures is mostly composed of DZP, besides small amounts of ingredients of DZP injection such as benzoic acid and benzyl alcohol.

At present, we cannot rule out the possibility that precipitation will occur after dilution of DZP injection with infusion fluids at any dilution ratio.
